# Comparative Proteomics Identified Proteins in Mung Bean Sprouts Under Different Concentrations of Urea

**DOI:** 10.3390/molecules30153176

**Published:** 2025-07-29

**Authors:** Lifeng Wu, Chunquan Chen, Xiaoyu Zhou, Kailun Zheng, Xiaohan Liang, Jing Wei

**Affiliations:** Key Laboratory of Tropical Fruits and Vegetables Quality and Safety, Institute of Food Testing, Hainan Academy of Inspection and Testing, State Administration for Market Regulation, Haikou 570311, China; 18976336989@163.com (L.W.); ccqccq2023@163.com (C.C.); zhouxiaoyu20200910@163.com (X.Z.); zhengkailun1997@163.com (K.Z.)

**Keywords:** comparative proteomics, mung bean, urea

## Abstract

Mung bean (*Vigna radiate*) sprouts are a popular choice among sprouted vegetables in Asia. Currently, the impact of nitrogen sources on the growth of mung bean sprouts remains poorly understood, and the underlying biological mechanisms responsible for the observed nonlinear growth patterns at different nitrogen levels have yet to be elucidated. In this research, in addition to conventional growth monitoring and quality evaluation, a comparative proteomics method was applied to investigate the molecular mechanisms of mung bean in response to 0, 0.025, 0.05, 0.075, and 0.1% urea concentrations. Our results indicated that mung bean sprout height and yield increased with rising urea concentrations but were suppressed beyond the L3 level (0.075% urea). Nitrate nitrogen and free amino acid content rose steadily with urea levels, whereas protein content, nitrate reductase activity, and nitrite levels followed a peak-then-decline trend, peaking at intermediate concentrations. Differential expression protein analysis was conducted on mung bean sprouts treated with different concentrations of urea, and more differentially expressed proteins participated in the L3 urea concentration. Analysis of common differential proteins among comparison groups showed that the mung bean sprouts enhanced their adaptability to urea stress environments by upregulating chlorophyll a-b binding protein and cationic amino acid transporter and downregulating the levels of glycosyltransferase, L-ascorbic acid, and cytochrome P450. The proteomic analysis uncovered the regulatory mechanisms governing these metabolic pathways, identifying 47 differentially expressed proteins (DEPs) involved in the biosynthesis of proteins, free amino acids, and nitrogen-related metabolites.

## 1. Introduction

With a history spanning more than 2000 years in Chinese culinary tradition, mung bean sprouts (*Vigna radiate*) remain a popular choice among sprouted vegetables in Asia [1,2]. Valued for their health benefits, these sprouts are frequently used in salads, sandwiches, or as a refreshing pre-meal appetizer [3]. Mung bean sprouts are known for heat-cleansing, energizing, and diuretic properties, as well as aiding in elimination and fat loss [4,5]. Contemporary research has validated mung bean sprouts as a functional food, supplying high-quality plant proteins, phenolic phytochemicals, essential lipids, sterols, carbohydrates, organic acids, amines, and growth-regulating hormones [1,6,7].

Urea serves as the major nitrogen-containing metabolite resulting from protein degradation in mammalian organisms and is usually used as a nitrogen fertilizer for plants, often referred to as a “life element” [8,9], accounting for 67% of the total agricultural N supply in China [10]. Driven by profit, unscrupulous merchants used urea in the production of mung bean sprouts to increase the yield and shorten the production cycle, resulting in “instant bean sprouts” [11,12,13,14,15]. Although the “National food safety standard—Maximum residue limits for pesticides in food” (GB 2763-2021) does not include urea [16], urea and other illegal additives soaked “problem bean sprouts” cannot be ignored. In recent years, the results of the State Administration of Market Supervision on the bean sprout sampling have shown that urea and other non-food ingredients containing bean sprouts continue to appear; the content of urea in mung bean sprouts was detected to be between 10 mg/kg and 500 mg/kg. The food safety of bean sprouts has garnered close attention.

Initial experimental trials revealed a pronounced phytotoxic effect on mung bean (*Vigna radiata*) sprout development when urea concentrations surpassed the 0.10% (*w*/*v*) threshold, manifesting as both germination suppression and growth retardation. Excessive urea can lead to ammonia toxicity, oxidative stress, and impaired protein synthesis [17]. It was found that a one-time application of nitrogen fertilizer had a nitrogen-limiting effect on mung beans, which severely restricted the growth and yield of the beans [18]. Additionally, urea-derived nitrogen significantly affects nitrate assimilation, free amino acid accumulation, and enzyme activities such as nitrate reductase (NR), which regulate nitrite and nitric oxide signaling pathways.

As fundamental macromolecules mediating cellular functions, proteins participate in nearly all biological processes [19]. Proteomic analysis enables systematic, large-scale investigations of cellular protein profiles, offering holistic insights into protein expression dynamics and functional roles under defined environmental parameters [20]. Numerous proteomic investigations have elucidated stress-responsive protein regulation in plants exposed to various biotic challenges [21,22,23,24]. However, there have been no studies analyzing differentially expressed proteins in mung bean sprouts treated with different concentrations of urea to elucidate the role of urea stress on proteins by proteomics.

In this study, we employed proteomics technology to analyze concentration-dependent growth responses, nutrient partitioning, and protein expression patterns in mung bean sprouts across a urea gradient (0–0.1%). Through a comprehensive investigation of quality parameter changes, we elucidated the functional roles of key proteins in mung bean sprout development. The findings of this research will address critical knowledge gaps regarding background urea levels in mung bean sprouts, providing scientific evidence to support regulatory authorities in establishing future food safety standards for urea content.

## 2. Results and Discussion

### 2.1. Quality Traits of Mung Bean Sprout

**Figure 1:**
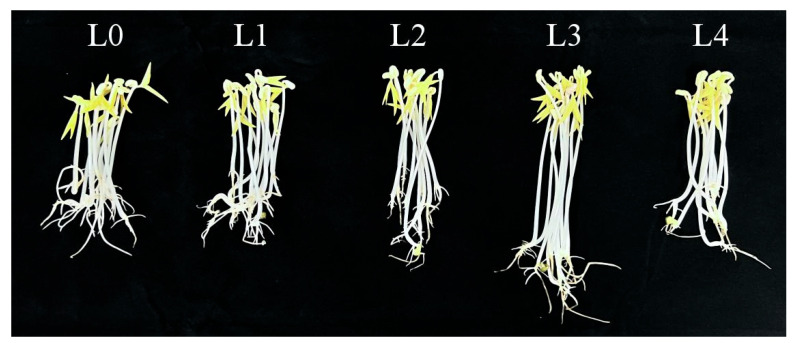
Growth of bean sprouts cultured for 7 days.

**Figure 2:**
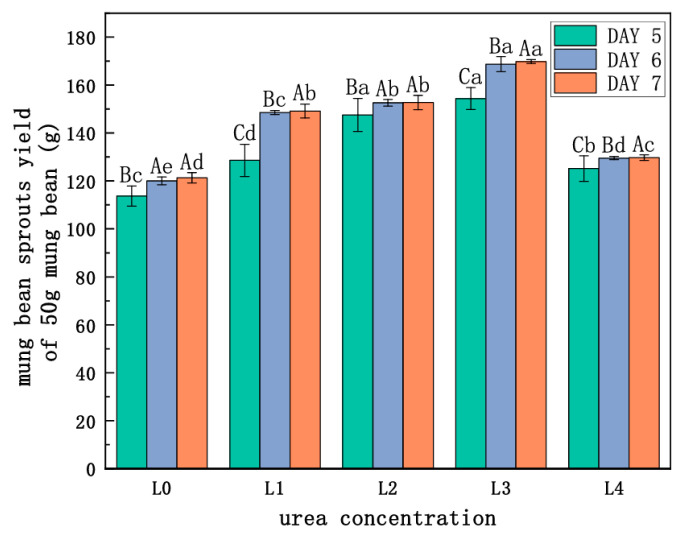
Effect of urea on the yield of mung bean sprouts. Note: different lowercase letters indicate significant differences at the same incubation time with different concentrations of urea (*p* < 0.05). Different capital letters represent significant differences between groups treated with the same concentration of urea at different incubation times (*p* < 0.05).

As Figure 1 and Figure 2 and Table 1 show, the green bean sprouts grown on the seventh day were the highest and heaviest at the L3 concentration and were significantly different compared to the other concentrations. It indicates that the yield and the length of bean sprouts increased with higher urea concentration when the urea concentration was lower than L3. However, the growth of bean sprouts was inhibited when the urea concentration exceeded L3. The use of urea had a significant effect on increasing the yield of mung bean sprouts at certain concentrations. Under L3 treatment, the highest yield of mung bean sprouts was recorded at 169.83 g on the seventh day, representing a 40% increase compared to freshwater control. Under L4, bean sprout yield decreased by 30.9% compared to the L3 level of treatment and increased only by 9.0% as compared to the control. Under the same urea level treatment, significant differences were observed on the sixth day compared to the fifth day. The results indicated that excessive use of urea was not effective in increasing the yield of mung bean sprouts.

Nitrogen is an essential element for plant growth and development, and the nitrogen content of plants generally ranges from about 0.3% to 5% of the plant’s dry weight [25]. Although nitrogen fertilizer plays an important role in plant growth and development, the application of nitrogen fertilizer in current agricultural production far exceeds the needs of plants [26]. Hodge [25] showed that plants can only absorb 30~50% of the applied nitrogen, and excessive nitrogen is detrimental to the growth of plants, which usually undergo adaptive changes in morphology, physiology, and metabolism to alleviate the nitrogen stress-induced injury [21]. Therefore, the excessive input of N fertilizer not only does not improve crop yield and quality but also leads to a reduction in N fertilizer utilization efficiency and an increase in N fertilizer loss, which causes serious environmental problems.

### 2.2. Nitrogen Composition of Mung Bean Sprout

This investigation examined key quality parameters of mung bean sprouts, focusing on protein profile, free amino acids, urea levels, soluble solids, and nitrite concentration, as shown in Table 2. With the urea concentration increasing from L0 to L3, the content of protein, urea, and nitrate increased 65.0%,214.8%, and 225.7%, respectively. As compared to L3, the content decreased by 21.8%, 1.3%, and 62.5% in L4. The free amino acids showed a tendency to increase, while the control had the lowest content of free amino acids.

During the germination of mung beans, proteolytic enzymes break down stored proteins, leading to a decline in protein content while increasing amino acids and non-protein nitrogen levels. This enzymatic activity is most pronounced during germination, as proteases continuously degrade insoluble macromolecular proteins into soluble small-molecule proteins or free amino acids [22]. These liberated amino acids are then transported to the developing embryo, where they serve as building blocks for synthesizing new proteins [23]. Consequently, the measured protein content in mung bean sprouts reflects both residual storage proteins and newly synthesized proteins. Combined with the results of protein content in Table 2, it shows that a certain concentration of exotic urea could lead to protein synthesis in mung bean sprouts.

The types and proportions of amino acids change continuously during the germination process, on the one hand, as certain amino acids are re-synthesized into new proteins with the growth of the sprouts, and on the other hand, the original proteins are used as a source of energy, especially in the early stages of seed germination. In addition, certain amino acids are readily catabolized during the soaking phase of the seed before germination, which is another potential factor in the continuous changes in the amino acid composition of proteins of the seed during germination [27]. As the results shown in Table 2 indicate, the changing tendency of protein and amino acids, except for exotic urea, could affect the content of amino acids.

During the production of mung bean sprouts, the nitrite content of bean sprouts without urea application was negligible (as shown in Table 2), and the nitrite content increased with the concentration of urea application. When the concentration of urea was 1%, it was close to the nitrite content of bean sprouts sold in the city of Foshan, which was in line with the limited standard of pollution-free vegetables, a view that was consistent with this finding [28]. According to the maximum limit standard in China (GB 2762-2022) [29], the limit of nitrite in vegetables is 20 mg/kg, and the nitrite concentration of bean sprouts in all the test groups in this study did not exceed the limit of food pollutants in China.

The determination of soluble solids in mung bean sprouts can partly reflect the breakdown of nitrogenous compounds and their subsequent assimilation into metabolic pathways. The soluble solids content within mung bean sprouts did not change with increasing concentrations of urea application, which might be associated with cells maintaining osmotic homeostasis by dynamically adjusting soluble solute concentrations [30]. However, both were significantly higher than the control (3.9% vs. 3.3%), indicating that the exotic urea could increase the content of soluble solids.

The results indicated that, except for the soluble solids, different concentrations of urea had a certain effect on the intrinsic quality of mung bean sprouts, especially on the nitrite content, urea content, and free amino acid content. The concentration of urea did not have a linear relationship with the quality of mung bean sprouts, suggesting that the plant body regulates through a variety of enzymes to ensure mung bean sprouts sprout and grow normally.

### 2.3. Identification and Function of Differentially Expressed Proteins (DEPs)

#### 2.3.1. Protein Identification and Quantification

**Figure 3:**
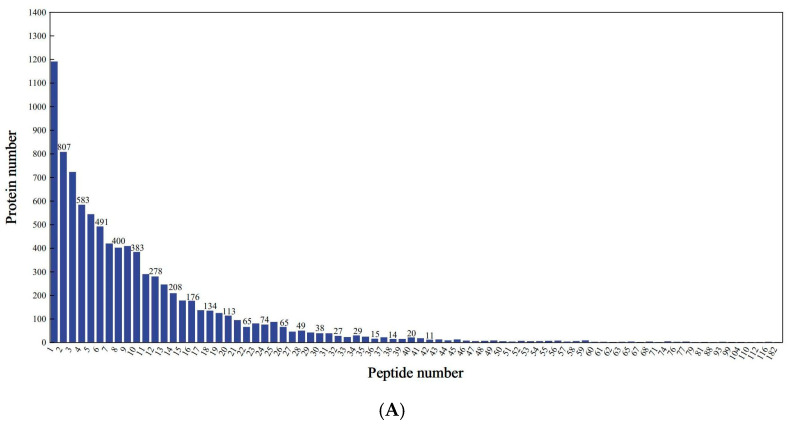

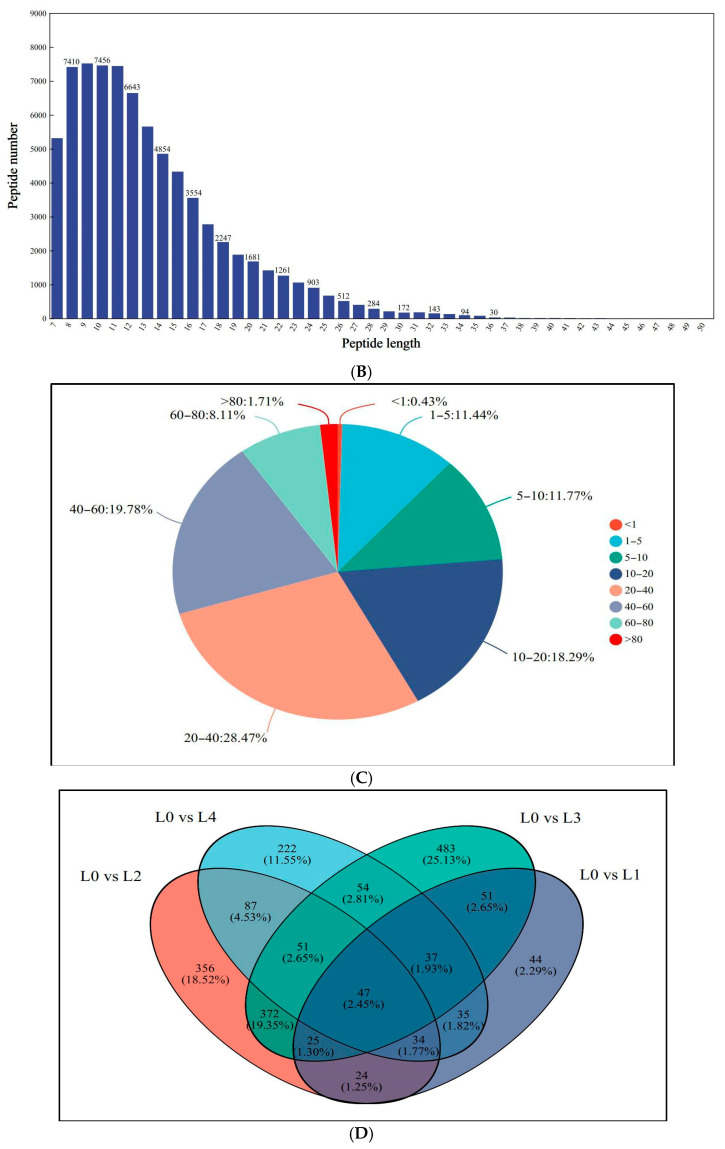
Identification and analysis of protein. (**A**) Peptide number distribution; (**B**) peptide length distribution; (**C**) protein coverage distribution; (**D**) shared differential protein Wayne plots.

Proteomic profiling identified 7372 distinct proteins across all treatment groups, corresponding to 82,066 peptide spectra matched to the mung bean proteome. As shown in Figure 3A,B, the peptide lengths ranged from 7 to 20 amino acids by extraction and detection of proteins. Analysis revealed that 89.75% of identified proteins exhibited sequence coverage exceeding 10% (Figure 3C), demonstrating the methodological robustness of the proteomic approach. Comparative assessment of protein profiles showed limited conservation, with merely 47 common proteins (2.45% of the total) present in all concentrations (Figure 3D). The majority of 1875 proteins (97.55% of the total) displayed sample-specific expression patterns, including 44 unique to L1, 356 to L2, 483 to L3, and 222 to L4. The changes in proteomics may lead to variations associated with the quality of mung bean sprouts. As protein expression influences mung bean sprout quality, key proteins may act as reliable quality markers.

#### 2.3.2. Differentially Expressed Proteins and Correlations

**Figure 4:**
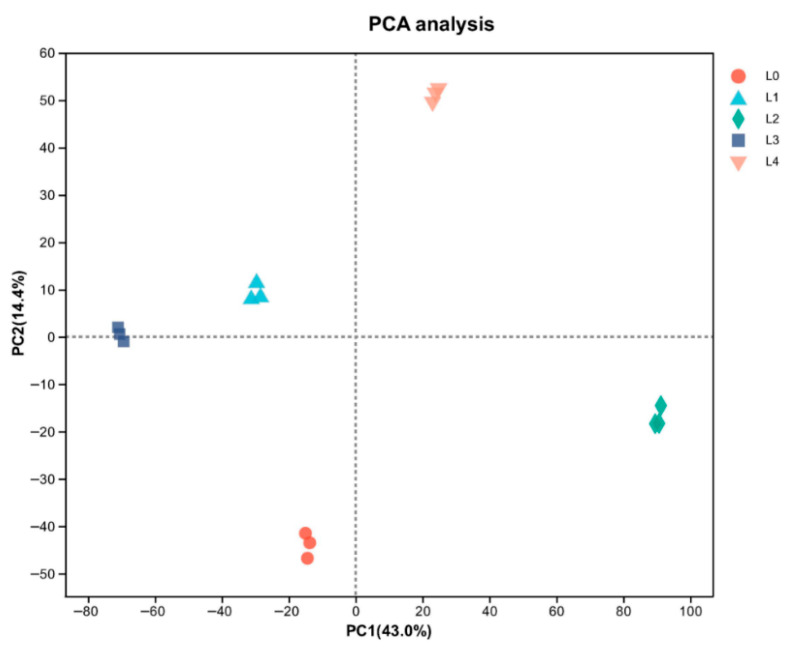
Principal component analysis (PCA) analysis of mung bean proteomic profiles.

**Figure 5:**
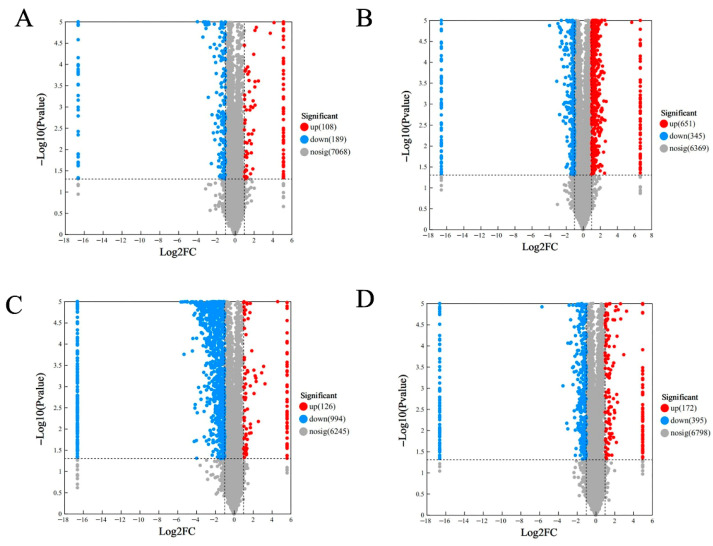
Volcanic diagram of differentially expressed proteins. (**A**) L0 vs. L1; (**B**) L0 vs. L2; (**C**) L0 vs. L3; (**D**) L0 vs. L4.

PCA demonstrated clear separation among proteomic profiles, with principal components explaining 43.0% (PC1) and 14.4% (PC2) of variance (Figure 4). The distinct clustering of all five concentrations suggests their protein expression patterns fundamentally differ, with DEP profiling providing critical insights into observed quality variations. Principal component analysis revealed distinct, non-overlapping clustering of all five concentrations, as shown in Figure 4. Understanding the profile of DEPs in the five mung bean concentrations is crucial for explaining their observed quality differences. Among the DEPs, illustrated in Figure 5, there were 297 (108 upregulated and 189 downregulated), 996 (651 upregulated and 345 downregulated), 1120 (126 upregulated and 994 downregulated), 567 (172 upregulated and 395 downregulated) proteins at L1, L2, L3, and L4, compared with L0, respectively. A total of 47 DEPs were shared proteins among the four groups. More proteins were changed in L2 and L3. These findings demonstrated that urea concentration significantly influences functional protein expression patterns.

#### 2.3.3. Enrichment Analysis of DEPs

**Figure 6:**
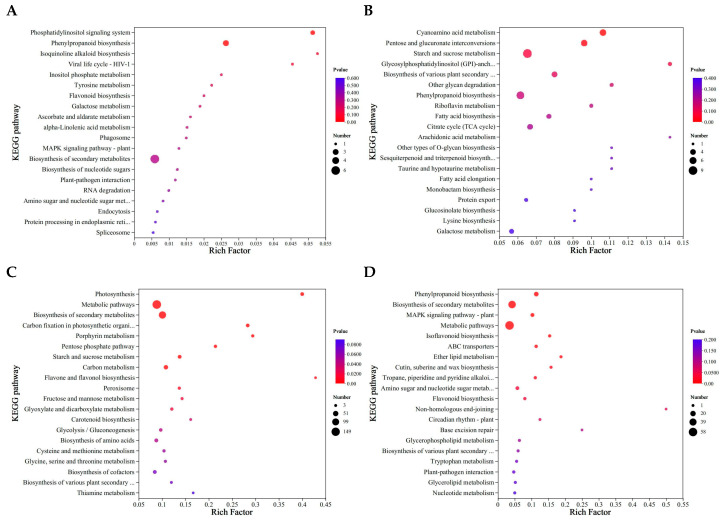
KEGG enrichment of differential proteins in mung bean sprouts. (**A**) L0 vs. L1; (**B**) L0 vs. L2; (**C**) L0 vs. L3; (**D**) L0 vs. L4.

Furthermore, as shown in Figure 6, the DEPs were classified by KEGG enrichment analysis to reveal their potential functions under different urea treatments. The quantity of identified pathways correlated significantly with the number of DEPs. Most DEPs in L0 vs. L1 were significantly enriched in isoquinoline alkaloid biosynthesis, phosphatidylinositol signaling system, viral life cycle-HIV-1,and phenylpropanoid biosynthesis (Figure 6A). The DEPs in L0 vs. L2 enrichment pathways were different from L0 vs. L1, which included arachidonic acid metabolism, Glycosylphosphatidylinositol (GPI)-anchor biosynthesis, other glycan degradation, other types of O-glycan biosynthesis, sesquiterpene and triterpene biosynthesis, taurine and hypotaurine metabolism, and cyanoamino acid metabolism (Figure 6B). The DEPs in L0 vs. L3 were specifically enriched in flavonoid and flavonol biosynthesis, photosynthesis, porphyrin metabolism, and carbon fixation in photosynthetic organisms (Figure 6C). The DEPs in L0 vs. L4 were specifically enriched in the non-homologous end joining, base excision repair, and ether lipid metabolism, with phenylpropanoid biosynthesis enriched most significantly (Figure 6D). The number of enrichment pathways was highest in L0 vs. L2. Consequently, special emphasis is placed on examining distinct and overlapping DEPs along with their regulated metabolic pathways in the subsequent analysis.

#### 2.3.4. GO Analysis of DEPs Under Different Concentrations of Urea

**Figure 7:**
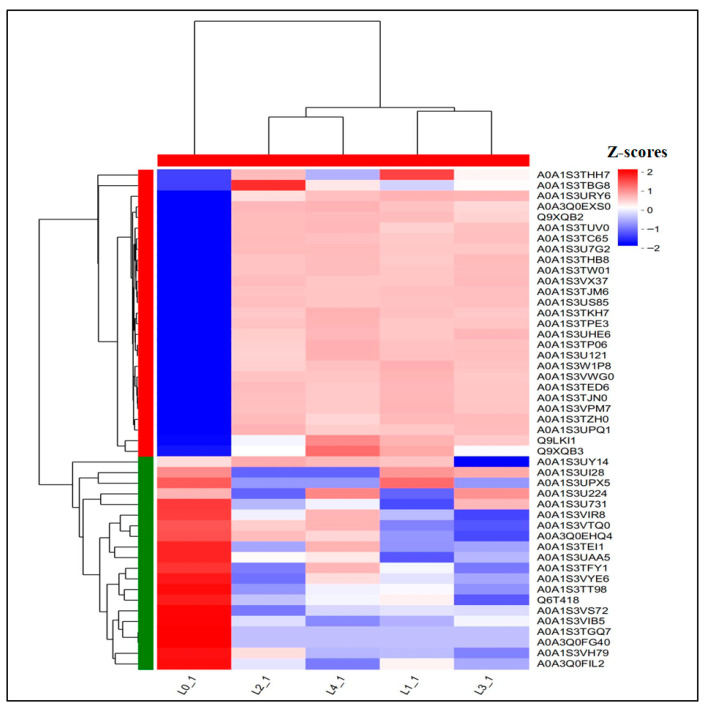
Heat map of shared DEPs.

The Wayne plot (Figure 3D) analysis of the shared DEPs showed that 47 proteins were differentially expressed across 5 concentrations. As shown in Figure 7 and Table 3, there were 27 proteins upregulated and 20 proteins downregulated in the total DEPs, in which the downregulated multiplicity of glycosyltransferase content was decreased by 2.5, 7.0 × 10^4^, 7.0 × 10^4^, and 7.0 × 10^4^-fold as compared to the control; the downregulated multiplicity of L-ascorbate oxidase content was 3.07, 2.40, 2.52, and 2.21-fold, respectively; and the downregulated multiples of patatin content were all 7.0 × 10^4^-fold compared to the control.

**Figure 8:**
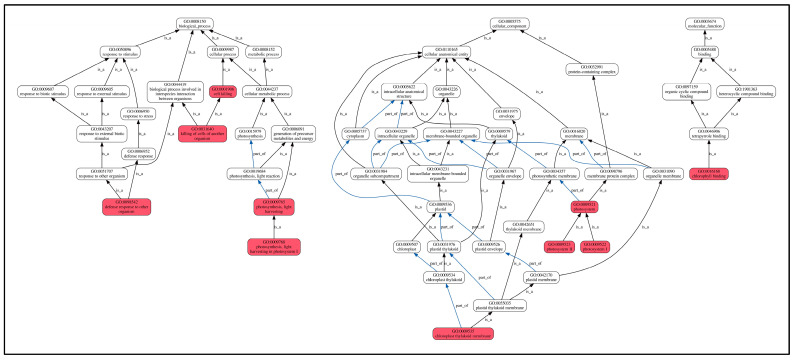
GO enrichment of shared DEPs. Note: the highlighted red box indicates significantly enriched GO terms. Lines between GO terms represent their relationships. The black arrows are shown as “is a”, where the direction indicates a hierarchical inclusion relationship (e.g., term A is a term B means A is a subtype of B). The blue arrows are shown as “part of”, where “C part of D” implies that if C occurs, it must be a component or part of D.

Furthermore, GO enrichment analysis (Figure 8) revealed that these shared DEPs were mainly enriched in biological processes (GO-BP) in defense responses to other organisms, killing cells of another organism, photosynthesis. The enriched proteins included cysteine-rich receptor-like protein kinase 10, NDR1/HIN1-like protein 12, phyto-defensins, defensin-like proteins, photosystems, and seven other proteins. Cellular components (GO-CC) were mainly enriched in chloroplast cyst-like membranes, photosystems I, photosystems II, and photosystems, with four enriched proteins, including photosystem I reaction center subunit IV and chlorophyll a-b binding protein. The proteins significantly enriched in molecular function (GO-MF) categories were predominantly associated with chlorophyll binding activity, including chlorophyll a-b binding proteins along with three additional chlorophyll-associated proteins. Chlorophyll biosynthesis serves as a key indicator of photosynthetic potential, and its upregulation directly enhances plant growth through increased light harvesting and carbon fixation. In this study, as shown in Table 3, compared to the control L0, all chlorophyll-related proteins showed an upregulating trend, which is one of the reasons for the significant growth compared to the control group.

Proteomic analysis offers a systematic approach to quantitatively examine protein-level metabolic responses in mung beans grown under varying urea concentrations. The dynamic cellular growth and metabolic changes are significantly influenced by differential urea availability. Previous research demonstrates that nutrient deficiency disrupts essential biosynthetic pathways, and this study proposes that altered urea levels may similarly induce metabolic imbalances in anabolic processes [31]. While energy imbalance occurs, metabolic disruption only partially inhibits growth. Mung beans transition from exponential growth to stabilization, during which urea stress upregulates galactose metabolism to produce myo-inositol galactosides. These metabolites serve dual protective roles: (1) osmoregulation via hydrophilic stabilization of proteins/membranes and (2) ROS detoxification under urea stress [32,33,34,35].Nitrogen limitation inhibits both cell division and lipid breakdown in early growth phases, promoting lipid storage. During maturation, cell proliferation ceases while lipid synthesis intensifies through enhanced fatty acid generation [34].

Betaine Aldehyde Dehydrogenase 1 (BADH1) catalyzes the oxidation of betaine aldehyde to glycine betaine (GB), a critical osmoprotectant that enhances plant stress tolerance. While GB accumulation is essential for osmoregulation and redox homeostasis, studies have proved that excessive GB may suppress cell division [36]. In this context, the observed downregulation of BADH1 could serve as a regulatory mechanism to maintain metabolic balance between stress adaptation and developmental processes, preserving photosynthetic efficiency to sustain carbon fixation [37]. The downregulation of BADH1 may optimize carbon fixation efficiency, thereby promoting plant growth. As a key catalytic enzyme in porphyrin metabolism, primarily through its heme-dependent redox activity, cytochrome P450 enzymes participate in modifying either the porphyrin ring or its side chains [38]. The downregulation of cytochrome P450 may reduce porphyrin biosynthesis, reducing ROS accumulation, which can protect photosynthetic apparatus and promote plant growth.In this study, the maximal growth of the L3 group may be correlated with (i) minimal BADH1 downregulation (avoiding GB overaccumulation), (ii) cytochrome P450 suppression (reducing ROS), and (iii) upregulated chlorophyll-binding proteins (boosting photosynthesis).

The membrane transport of amino acids in plant systems involves three key transporter categories: specific amino acid carriers, polyamine-associated transporters, and multi-substrate extrusion pumps [39]. Alterations in amino acid transporter expression may disrupt amino acid balance in plants, subsequently influencing metabolic pathways and developmental processes. Notably, proline transport and cellular accumulation serve as protective mechanisms against environmental stresses [40,41]. Under saline and drought conditions, *Arabidopsis thaliana* exhibited significantly higher proline accumulation compared to wild-type specimens [42]. The current investigation revealed significant upregulation of cationic amino acid transporters in comparison to control groups, indicating their protective role against urea stress in mung bean sprouts. This enhanced transporter activity correlated with increased proline accumulation across L1–L4 urea concentrations.

L-ascorbic acid (AsA) is one of the important antioxidant molecules and plays an important role in cell division, expansion, and plant growth. Glucose is converted to AsA through five intermediate compounds, which are considered to be the major AsA biosynthesis pathway in plants [43]. In plants, the AsA cycling pathway (ascorbate-glutathione system) plays an important role in stress response and adaptation [44]. High nitrogen concentration has been reported to reduce AsA levels in vegetables [45], and short-term low nitrogen treatment significantly increased ascorbic acid levels in cucumbers [46]. In the present study, it was found that L-ascorbate oxidase content was downregulated in the urea-treated group as compared to the control group, which may be attributed to the fact that ascorbate oxidase can oxidize ascorbic acid under urea-stressed conditions, leading to a reduction in reactive oxygen species and benefiting the growth of bean sprouts [47].

Cytochrome P450s (CYPs) belong to the oxidoreductase class of enzymes, one of the largest families of enzymes containing heme thioesters as cofactors, which can act as functional catalysts and play a crucial role in the synthesis of antioxidants and phytohormones in plants [48]. Cytochrome P450 is also directly involved in plant secondary metabolism, acting as a detoxifier of metabolic byproducts generated in response to external compounds or stress [49,50]. In addition, cytochrome P450 is involved in protecting plants from harsh environmental conditions by enhancing the activity of compounds with antioxidant properties, such as flavonoids [51,52]. It has been reported that the CYP71A25 and CYP71B2 genes are upregulated under drought stress in sorghum plants [49]. In this study, the downregulation of cytochrome P450 content in the urea-treated group compared to the control group reduced the activity of antioxidant compounds, thus affecting the growth of bean sprouts.

## 3. Materials and Methods

### 3.1. Urea Treatment and Culture Conditions

Mung beans were purchased from the market in Haikou, China. Twenty-five grams of mung beans were immersed in five different urea concentrations overnight: 0% (L0), 0.025% (L1), 0.05% (L2), 0.075% (L3), and 0.1% (L4) (three replicates for each urea concentration).

### 3.2. Mung Bean Sprouts and Culture Conditions

The immersed mung beans were placed in a 25 °C incubator with a glass window, and the bottom was covered with two layers of filter paper to keep the seeds in a moist environment. Mung bean sprouts were rinsed with distilled water at 12 h intervals and harvested after 7 d in the incubator in October 2023. Freshly harvested samples were stored at 4 °C for physicochemical analysis within 24 h, while others were stored in the refrigerator at −80 °C for subsequent proteomic studies.

### 3.3. Analysis Method

Nitrite content was analyzed by GB 5009.33-2016 (Standardization Administration of China, 2016), using the naphthylenediamine hydrochloride method by ultraviolet spectrophotometer (2600i, Shimazu, Tokyo, Japan) [53] at 538 nm. The urea content was assayed by high-performance liquid chromatography (5500, AB SCIEX, Framingham, MA, USA) according to our previously validated method [54]. The soluble solids were measured by NY/T 2637-2014 (Ministry of Agriculture of the People’s Republic of China, 2014) using an Abbe Refractometer (WYA-2S, Bell, Xi’an, China) [55] at 20 °C, and values were recorded as a percentage. The protein was analyzed using the AOAC 992.15 method, determining Kjeldahl nitrogen (KND) [56].

Free amino acid content was measured using a ninhydrin colorimetric assay with an ultraviolet spectrophotometer (2600i, Shimazu, Japan), according to the reference Hung et al. with minor modifications [57]. Ten grams freshly mung bean sprouts were weighed and diluted to 100 mL with distilled water. The mixture was vortexed for 30 min and then filtered using a membrane filter (0.45 μm).The ninhydrin reaction was carried out by mixing 3.0 mL of the filtrate with 1.0 mL sodium acetate buffer (pH 5.0) and 2.0 mL of 2.0% ninhydrin solution in a 25 mL volumetric flask. After heating at 100 °C for 19 min and cooling, the volume was adjusted to 25 mL with distilled water, and absorbance was measured at 568 nm.

### 3.4. Total Protein Extraction

Total protein extraction was performed according to the method of Hsu et al. with minor modifications [17]. Frozen samples were placed on ice, then suspended in protein lysis buffer (8 M urea, 1% SDS) supplemented with protease inhibitors. The mixtures were homogenized using a high-throughput tissue grinder (3 cycles, 40 s/cycle). The mixture was incubated on ice for 30 min with intermittent vortex mixing (5–10 s every 5 min). Following high-speed centrifugation (16,000× *g*, 30 min at 4 °C), the soluble protein fraction was collected and quantified using the Bicinchoninic Acid (BCA) Assay Kit (Thermo Scientific, Waltham, MA, USA) according to the manufacturer’s protocol. After quantification, proteins were separated by SDS-PAGE (Thermo Scientific, Waltham, MA, USA).

### 3.5. Protein Digestion

Protein digestion was performed according to the method of Hsu et al. with minor modifications [17]. Protein samples (100 μg) were reconstituted in 100 mM triethylammonium bicarbonate (TEAB) buffer and then performed using 10 mM tris(2-carboxyethyl)-phosphine (TCEP) at 37 °C for 60 min, followed by alkylation with 40 mM iodoacetamide (IAM) in darkness at room temperature for 40 min. After centrifugation (10,000× *g*, 4 °C, 20 min), the pellet was collected and resuspended in 100 μL of 100 mM TEAB buffer. Trypsin digestion was carried out at a 1:50 (*w*/*w*) enzyme-to-protein ratio with overnight incubation at 37 °C.

### 3.6. Peptide Desalting and Quantification

Peptide desalting and quantification were performed according to the method of Sun et al. with minor modifications [31]. Digested peptides were concentrated by vacuum evaporation and then resuspended in 0.1% (*v*/*v*) trifluoroacetic acid. Sample cleanup was performed using hydrophilic-lipophilic balanced (HLB) SPE cartridges prior to solvent removal via SpeedVac centrifugation (Eppendorf, Hamburg, Germany). Final peptide concentrations were determined by peptide-specific colorimetric assay (Thermo Fisher Scientific Peptide Quantification Kit).

### 3.7. DIA Mass Detection

DIA mass detection was performed according to the method of Sun et al. with minor modifications [31]. Peptides were separated on a C18 column (75 μm × 25 cm, Ionopticks, Fitzroy, Australia) using solvent A: 2% ACN/0.1% FA; solvent B: 80% ACN/0.1% FA; with a 60-min gradient: 3–28% B (0–45 min) → 28–44% B (45–50 min) → 44–90% B (50–55 min) → 90% B (55–60 min), at 250 nL/min flow rate. The sample injection volume was 10 μL.

Data-independent acquisition (DIA) was performed on a timsTOF Pro 2 with an *m*/*z* range of 400–1200, an ion mobility range of 0.57–1.47 Vs/cm^2^, 100 ms accumulation/ramp time, 1 MS + 10 PASEF MS/MS scans per TIMS cycle, 0.4 min dynamic exclusion, and 64 DIA windows (25 Th each).

### 3.8. Protein Identification

DIA data were processed using Spectronaut software (Version 14) with iRT-aligned retention times, 6 peptides/protein and 3 transitions/peptide, strict FDR thresholds (protein/peptide ≤1%), high-confidence filters (peptide confidence ≥99%), precise quantification (XIC width ≤75 ppm). Quantification was performed by integrating peak areas of unique peptide transitions, with shared peptides and post-translationally modified peptides systematically excluded from analysis. Protein identification requires the detection of at least one unique proteotypic peptide.

### 3.9. Statistical Analyses

All experiments were conducted in triplicate, and the results were presented as mean ± standard deviation (SD). The one-way analysis of variance (ANOVA) and least significant difference (LSD) tests were used to identify significant variations (*p* < 0.05).

Statistical analysis of the proteomic data was conducted using the Majorbio Cloud platform (https://cloud.majorbio.com), and the “Uniprot database” was used for protein identity assignment and accession numbers. The proteomic data have been deposited in the iProX repository. Differential protein expression between experimental groups was assessed through *t*-tests implemented in R (https://www.r-project.org/), with the calculation of both *p*-values and fold change values. Differentially expressed proteins (DEPs) were identified using the following criteria: fold change >1.2 or <0.83 with statistical significance (*p* < 0.05). Functional characterization was performed through Gene Ontology (GO) (http://geneontology.org/) annotation and KEGG pathway analysis (http://www.genome.jp/kegg/, accessed on 25 october 2024). Subsequently, enrichment analyses were conducted for the DEPs using these databases.

## 4. Conclusions

This is the first study to elucidate the mechanisms by which comparative proteomics applies to the quality traits of mung bean sprouts under different concentrations of urea. Integrated analysis of morphological, biochemical, and proteomic data revealed that the L3 treatment elicited the most pronounced stress response, with a significant number of differentially expressed proteins (DEPs). Functional annotation (GO) and pathway analysis (KEGG) demonstrated that mung bean sprouts produced distinct protein profiles with varying protective functions under different urea concentrations. Comparative proteomic analysis of the four treatment groups indicated that low-nitrogen stress triggered adaptive responses through the upregulation of key proteins, including glycosyltransferases, cationic amino acid transporter proteins, L-ascorbic acid, cytochrome P450, betaine aldehyde dehydrogenase, potato glycoprotein patatin, and chlorophyll a-b-binding proteins. In this study, the downregulation of cytochrome P450 content in the urea-treated group compared to the control group reduced the activity of antioxidant compounds, thus affecting the growth of bean sprouts. The proteomic analysis uncovered the regulatory mechanisms governing these metabolic pathways, identifying 47 differentially expressed proteins (DEPs) involved in the biosynthesis of proteins, free amino acids, and nitrogen-related metabolites.

## Figures and Tables

**Table 1:** Effect of urea on height of mung bean sprouts.

Urea Concentration	Day 5 Height (cm)	Day 6 Height (cm)	Day 7 Height (cm)
L0	11.10 ^dB^ ± 0.15	13.75 ^cA^ ± 0.14	14.03 ^dA^ ± 0.06
L1	12.90 ^bC^ ± 0.22	15.40 ^bB^ ± 0.12	15.72 ^cA^ ± 0.14
L2	13.90 ^aB^ ± 0.08	15.55 ^bA^ ± 0.20	15.97 ^bA^ ± 0.23
L3	13.90 ^aC^ ± 0.14	16.00 ^aB^ ± 0.11	16.84 ^aA^ ± 0.17
L4	12.36 ^cC^ ± 0.11	12.58 ^dB^ ± 0.24	13.02 ^eA^ ± 0.24

Note: different lowercase letters indicate significant differences at the same incubation time with different concentrations of urea (*p* < 0.05). Different capital letters represent significant differences between groups treated with the same concentration of urea at different incubation times (*p* < 0.05).

**Table 2:** Effect of urea on internal quality of mung bean sprouts.

Urea Level	Protein Content (G/100 g)	Free Amino Acid Content (Mg/G)	Soluble Solids (%)	Urea Content (Mg/Kg)	Nitrite Content (Mg/Kg)
L0	2.06 ^e^ ± 0.08	5.044 ^e^ ± 0.122	3.3 ^b^ ± 0.1	2.218 ^e^ ± 0.044	0.327 ^c^ ± 0.006
L1	2.26 ^d^ ± 0.07	5.373 ^d^ ± 0.134	3.9 ^a^ ± 0.1	3.682 ^d^ ± 0.052	0.329 ^c^ ± 0.009
L2	2.54 ^c^ ± 0.10	5.734 ^c^ ± 0.055	3.9 ^a^ ± 0.1	3.912 ^c^ ± 0.032	0.431 ^b^ ± 0.011
L3	3.40 ^a^ ± 0.11	5.994 ^b^ ± 0.089	3.9 ^a^ ± 0.1	6.960 ^a^ ± 0.022	1.065 ^a^ ± 0.020
L4	2.66 ^b^ ± 0.05	6.419 ^a^ ± 0.102	3.9 ^a^ ± 0.1	6.883 ^b^ ± 0.045	0.399 ^bc^ ± 0.008

Note: different lowercase letters in the same column indicate significant differences between groups treated with different concentrations of urea (*p* < 0.05).

**Table 3:** Fold changes of partially shared DEPs.

Accession	Description	Fold Change log_2_^FC^			
		L0 vs. L1	L0 vs. L2	L0 vs. L3	L0 vs. L4
A0A1S3TC65	Cationic amino acid transporter 4	5.14	6.70	5.62	5.00
A0A1S3TFY1	Cytochrome P450 83B1	−1.95	−2.79	−2.66	−1.07
A0A1S3TT98	Betaine aldehyde dehydrogenase 1, chloroplastic	−1.02	−1.43	−1.51	−1.02
A0A1S3UAA5	L-ascorbate oxidase	−1.62	−1.26	−1.33	−1.15
A0A1S3UPX5	Glycosyltransferase	−1.33	−16.61	−16.61	−16.61
A0A3Q0FG40	Patatin	−16.61	−16.61	−16.61	−16.61
A0A3Q0FIL2	Seed linoleate 9S-lipoxygenase-3-like	−1.45	−1.60	−2.02	−2.42
Q6T418	Plant defensin	−1.39	−1.83	−2.78	−1.56
Q9LKI1	Chlorophyll a-b binding protein, chloroplastic	2.48	1.47	2.32	2.99
Q9XQB2	Chlorophyll a-b binding protein, chloroplastic	5.14	6.70	5.62	5.00
Q9XQB3	Chlorophyll a-b binding protein, chloroplastic	2.12	1.83	1.30	2.32

## Data Availability

The mass spectrometry proteomics data generated in this study have been deposited in the iProX repository under accession number PXD066373. (https://www.iprox.cn/page/project.html?id=IPX0012699000, accessed on 1 June 2025).
